# Amygdala enlargement and emotional responses in (autoimmune) temporal lobe epilepsy

**DOI:** 10.1038/s41598-018-27914-z

**Published:** 2018-06-22

**Authors:** Olga Holtmann, Insa Schlossmacher, Constanze Moenig, Andreas Johnen, Lisa-Marie Rutter, Jan-Gerd Tenberge, Patrick Schiffler, Judith Everding, Kristin S. Golombeck, Christine Strippel, Andre Dik, Wolfram Schwindt, Heinz Wiendl, Sven G. Meuth, Maximilian Bruchmann, Nico Melzer, Thomas Straube

**Affiliations:** 1 Institute of Medical Psychology and Systems Neuroscience, University of Muenster, Muenster, Germany; 2 Otto Creutzfeldt Center for Cognitive and Behavioral Neuroscience, University of Muenster, Muenster, Germany; 3 Department of Neurology, University of Muenster, Muenster, Germany; 4 Department of Clinical Radiology, University of Muenster, Muenster, Germany

## Abstract

Temporal lobe epilepsy with amygdala enlargement (TLE-AE) is increasingly recognized as a distinct adult electroclinical syndrome. However, functional consequences of morphological alterations of the amygdala in TLE-AE are poorly understood. Here, two emotional stimulation designs were employed to investigate subjective emotional rating and skin conductance responses in a sample of treatment-naïve patients with suspected or confirmed autoimmune TLE-AE (*n* = 12) in comparison to a healthy control group (*n* = 16). A subgroup of patients completed follow-up measurements after treatment. As compared to healthy controls, patients with suspected or confirmed autoimmune TLE-AE showed markedly attenuated skin conductance responses and arousal ratings, especially pronounced for anxiety-inducing stimuli. The degree of right amygdala enlargement was significantly correlated with the degree of autonomic arousal attenuation. Furthermore, a decline of amygdala enlargement following prompt aggressive immunotherapy in one patient suffering from severe confirmed autoimmune TLE-AE with a very recent clinical onset was accompanied by a significant improvement of autonomic responses. Findings suggest dual impairments of autonomic and cognitive discrimination of stimulus arousal as hallmarks of emotional processing in TLE-AE. Emotional responses might, at least partially, recover after successful treatment, as implied by first single case data.

## Introduction

Temporal lobe epilepsy with amygdala enlargement (TLE-AE) is increasingly recognized as a distinct adult electroclinical syndrome of heterogeneous aetiology^1^. It is often associated with autoantibody (aab)-positive limbic encephalitis (LE)^2,3^ but also occurs as a (yet) aab-negative condition sometimes associated with focal cortical dysplasias or tumors of the amygdala^4^. Clinically, TLE-AE is best described by (i) mesial temporal lobe epilepsy, (ii) declarative memory disturbance, and (iii) rather poorly characterized affective symptoms^1^. Magnetic resonance imaging (MRI) typically reveals characteristic uni- or bilateral enlargement and increases of T_2_/fluid attenuation inversion recovery (FLAIR) signal intensities of the amygdala (and the anterior hippocampus)^1–5^. Moreover, most patients display interictal epileptiform discharges (IEDs) ipsilateral to the enlarged amygdalae. The site of IEDs is known to correspond tightly to the seizure-onset zones in TLE suggesting the enlarged amygdalae as seizure focus in TLE-AE^6^. While the declarative memory disturbance is likely explained by co-affection of the hippocampus, predominant involvement of the amygdala has been suggested in some emotional changes in TLE-AE^7^. However, despite the promise of a strong clinical benefit, emotion processing in TLE-AE and its relation to morphological changes of the amygdala has not been addressed up to now.

Evidence from animal and human research suggests a pivotal role of the amygdala in emotion processing and in appropriate homoeostatic behaviour to emotionally salient stimuli^7,8^. Considering basic emotions, processing of negative emotions – especially of fear – has been particularly disturbed by amygdala damage of various aetiologies^9–12^. However, some studies report apparently normal emotion processing in patients with amygdala lesions^13,14^. Beyond this, the amygdala has been proved to be involved in neuroendocrine and autonomic functions, including skin conductance responses (SCRs) to emotionally arousing stimulation^15,16^. There is some scattered evidence that patients suffering from confirmed autoimmune TLE-AE show impaired autonomic arousal during demanding cognitive tasks^17^. In a previous single case study we found a strong impairment of SCRs to emotional film clips in a patient suffering from confirmed autoimmune TLE-AE as compared to healthy controls^18^. In contrast, subjective emotional responses were not significantly impaired in this patient. While this single case strongly suggests a pronounced autonomic dysfunction to emotional stimuli in confirmed autoimmune TLE-AE, single cases need to be interpreted with caution. To better characterize emotional and autonomic responses in TLE-AE, studies in a sample of suited patients with treatment-naïve TLE-AE are demanded. Furthermore, to better understand consequences of the neuropathology in TLE-AE, investigating the link between amygdala enlargement and emotional responses would be exceptionally informative. Finally, providing evidence that recovery of potential deficits might coincide with successful immunotherapy and decline of morphological alterations of the amygdala in both suspected and confirmed autoimmune TLE-AE would support the claim that emotional alterations (i) are not a premorbid condition but induced by TLE-AE, and (ii) are not an irreversible outcome but dependent on therapeutically tractable amygdala function.

In the current study, we investigated emotional and autonomic responses to emotional stimulation by means of emotion-inducing videos (experiment 1) and images (experiment 2) in a sample of treatment-naïve patients suffering from suspected and confirmed autoimmune TLE-AE in comparison to a matched healthy control group. Possible relations between autonomic and behavioural changes and structural amygdala alterations were addressed. Additionally, a subgroup of patients was investigated during the course of immunotherapy to examine whether emotional alterations are, at least partially, reversible.

## Methods

### Participants

**Figure 1:**
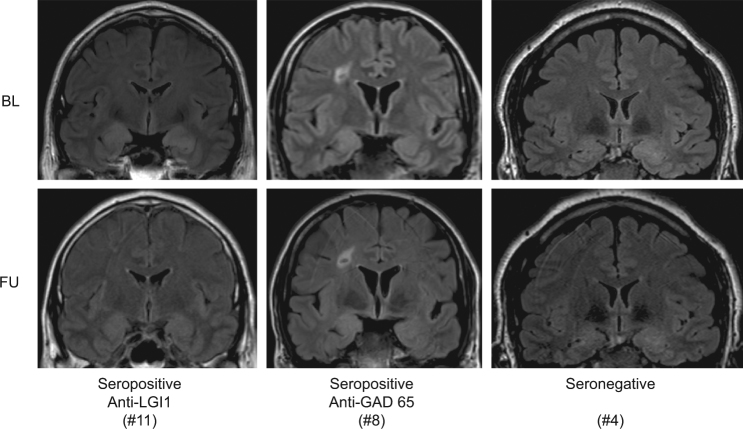
Representative FLAIR images at baseline (BL, upper panels) and follow-up (FU, lower panels) of confirmed autoimmune TLE-AE with anti-LGI1 aabs (Patient #11, left panels), anti-GAD65 aabs (Patient #8, middle panels), as well as suspected autoimmune TLE-AE (Patient #4, right panels) demonstrate uni- or bilateral hyperintensity and enlargement of the amygdala. Note that changes at BL were clearly regressive at FU in patient #11.

Twelve patients suffering from treatment-naïve suspected or confirmed autoimmune TLE-AE (three women, mean age ± standard deviation [SD]: 52.75 ± 14.56 years, age range: 27–73 years) participated in experiment 1. From this sample, eleven patients (two women, mean age ± SD: 54.45 ± 13.97, age range: 27–73 years) participated in experiment 2. All patients were recruited from the Department of Neurology, University of Muenster, Germany, and fulfilled criteria for TLE-AE: (i) temporal lobe epilepsy, declarative memory disturbance, and affective disturbance; and (ii) MRI data showing uni- or bilateral enlargement and T_2_/FLAIR signal hyperintensity of the amygdala (and the anterior hippocampus)^1^ (Fig. 1). All patients showed inflammatory CSF changes consisting of lymphocytic pleocytosis (LP), and/or elevated fractions of activated HLADR^+^ CD4^+^ T cells and/or elevated fractions of activated HLADR^+^ CD8^+^ T cells (aTCs) and/or elevated fractions of activated CD19^+^ CD138^+^ B cells/plasma cells (aBCs) compared to an age-matched control population and/or ≥3 CSF-specific oligoclonal bands (OCBs) consistent with a neuroinflammatory origin of TLE-AE (for clinical data see Table 1). Sera and CSF analyses identified four patients positive for Leucine-rich, glioma inactivated 1 (LGI1) aabs, one patient positive for contactin-associated protein 2 (CASPR2) aabs, and two patients positive for glutamate decarboxylase 65 (GAD65) aabs (confirmed autoimmune TLE-AE). In five patients, none of the hitherto known aabs could be detected (suspected autoimmune TLE-AE). Immunotherapeutic treatment for suspected and confirmed autoimmune neuroinflammation consisted of intravenous methylprednisolone pulse therapy (MPPT; 1 g/day on five consecutive days) with oral taper together with immunoadsorption (IA; total of five sessions on consecutive days with treatment of at least two plasma volumes). Steroid sparing agents such as azathioprine (AZA), methotrexate (MTX), or rituximab (RTX) were initiated depending on the individual severity of the clinical syndrome. In addition, patients received anticonvulsive and antidepressant treatments. Comprehensive neuropsychological assessment targeting major neurocognitive domains was conducted, with emphasis on memory and attention (Table 2). In addition to the investigation at first clinical presentation (baseline, BL), five patients were observed three (follow-up 1, FU1) resp. six months (follow-up 2, FU2) after their initial immunotherapeutic treatment.

**Table 1:** Single case demographical and clinical data of patients.

Patient	Age/gender	Disease duration at BL	Symptoms at BL	Side of AE on MRI and IED on EEG	Time of measurement	CSF infla-mmation	Autoantibody titer serum/CSF	Immuno-therapy	Medication
1	34/F	11 years	TLE, MD, AD	left, temporal left	BL	aTCs	unknown	IA, Steroids iv	CBZ, ZON
					FU1	—	—	—	—
					FU2	—	—	—	—
2	65/M	4 months	TLE, MD, AD	bilateral (right > left), bitemporal (right > left)	BL	LP, aBCs, aTCs	VGKC (CSF 189 pmol/l, Serum 410 pmol/l) CASPR2 (CSF 1:320, Serum 1:3200)	IA, Steroids iv	LEV
					FU1	LP, aBCs, aTCs	n.d.	IA, Steroids iv	LEV
					FU2	—	—	—	—
3	57/M	5 years	TLE, MD, AD	bilateral (left > right), bitemporal (left > right)	BL	LP, aTCs	unknown	IA, Steroids iv	LEV
					FU1	—	—	—	—
					FU2	—	—	—	—
4	27/M	2 years	TLE, AD	left, temporal (left)	BL	aTCs	unknown	IA, Steroids iv	LTG, DUL
					FU1	n.d.	n.d.	IA, Steroids iv	LTG, DUL
					FU2	—	—	—	—
5	73/M	7 months	TLE, MD, AD	bilateral (right > left), temporal (right > left)	BL	aTCs	unknown	Steroids iv	LEV
					FU1	—	—	—	—
					FU2	—	—	—	—
6	43/M	19 years	TLE, AD	bilateral (left > right), bitemporal (left > right)	BL	aTCs	GAD65 (CSF 1:32, Serum 1:320)	—	FBM, ESC, AMT
					FU1	n.d.	n.d.	—	FBM, ESC, AMT
					FU2	n.d.	n.d.	—	—
7	58/M	2 years	TLE, MD, AD	bilateral (right > left), bitemporal (right > left)	BL	aTCs	LGI1 (CSF positive, Serum positive)	IA, Steroids iv, AZA	LTG, QTP
					FU1	—	—	—	—
					FU2	none	n.d.	AZA	LTG, QTP
8	48/F	2 months	TLE, MD	right, temporal right	BL	aTCs, OCB	GAD65 (CSF 1:100, Serum 1:1000)	Steroids iv	LEV
					FU1	aTCs	GAD65 (CSF 1:100, Serum 1:3200)	Steroids iv	LEV
					FU2	n.d.	n.d.	Steroids iv	LEV
9	42/M	2 months	TLE, MD	bilateral (right > left), bitemporal (right > left)	BL	aTCs	unknown	Steroids iv	LEV
					FU1	—	—	—	—
					FU2	—	—	—	—
10	66/M	2 years	TLE, AD	bilateral (left > right), bitemporal (left > right)	BL	LP, aBCs	VGKC (CSF positive, Serum positive) LGI1 (CSF 1:3.2, Serum negative)	IA, Steroids iv, AZA	LEV, LCM
					FU1	aBCs	VGKC (CSF positive, Serum positive) LGI1 (CSF 1:3.2, Serum negative)	Steroids iv, AZA	LEV, LCM
					FU2	n.d.	n.d.	AZA	LEV, LCM
11	50/M	1 month	TLE, MD, AD	bilateral (left > right), bitemporal (left > right)	BL	LP, aTCs	LGI1 (CSF negative, Serum 1:32)	IA, Steroids iv, RTX	LEV
					FU1	aTCs	n.d.	RTX	LEV
					FU2	n. d.	n.d.	RTX	LEV
12	70/F	6 months	TLE, MD	left, temporal left	BL	aBCs, aTCs	LGI1 (CSF negative, Serum 1:1000)	IA, Steroids iv	LEV
					FU1	aTCs	LGI1 (CSF 1:10, Serum 1:32)	IA, Steroids iv	LEV
					FU2	—	—	—	—

Abbreviations: aBCs: activated B cells; AE: amygdala enlargement; aTCs: activated T cells; AD: affective disturbance; AMT: agomelatine; AZA: azathioprine; CASPR2: contactin-associated protein-2; CBZ: carbamazepine; CyP: cyclophosphamide; DUL: duloxetine; EEG: electroencephalography; ESC: eslicabazepin; F: female; FBM: felbamate; GAD65: 65 kDa isoform of decarboxylase; IA: immunoadsorption; IED: interictal epileptic discharges; LCM: lacosamide; LEV: levetiracetam; LGI1: leucine-rich, glioma inactivated 1 protein; LP: lymphocytic pleocytosis; LTG: lamotrigine; M: male; n.d.: not determined; MD: memory disturbance; OCB: oligoclonal bands; PE: plasma exchange; QTP: quetiapine; RTX: rituximab; TLE: epilepsy with temporal lobe seizures; VGKC: voltage-gated potassium channel; ZON: zonisamide.

**Table 2:** Neuropsychological domain performance of the patient group.

Domain and subtests	Baseline				Follow-up			
	N	mean [range]	normative z-score [range]	% of patients impaired	N	mean [range]	normative z-score [range]	% of patients impaired
**Cognitive screening**								
Epitrack	9	32.56 [19; 39]	N/A^a^	22.2^a^	6	33.83 [27; 39]	N/A^a^	16.7^a^
**Verbal memory**								
**Verbal Span**								
VLMT trial 1	10	5.7 [3; 12]	−0.75 [−1.75; 1.75]	60	7	6 [2; 9]	−0.53 [−1.75; 0.84]	28.6
**Verbal learning**								
VLMT trial 1–5	10	43.2 [23; 67]	−0.56 [−1.75; 1.65]	40	7	43.57 [18; 67]	−0.53 [−1.75; 1.65]	28.6
**Verbal ST retrieval**								
VLMT trial 6	10	7.6 [2; 14]	−0.83 [−1.75; 0.84]	50	7	7.14 [0; 15]	−0.87 [−1.75; 1.04]	42.9
**Verbal LT retrieval**								
VLMT trial 7	9	7.22 [1; 15]	−0.76 [−1.75; 1.28]	55.6	7	7 [0; 14]	−0.85 [−1.75; 0.52]	28.6
**Verbal recognition**								
VLMT trial 8 true	9	12.67 [9; 15]	−0.38 [−1.75; 0.84]	22.2	7	12.29 [9; 15]	−0.62 [−1.28; 0]	0
**Visuospatial skills**								
**Visuoconstruction**								
RCFT copy	8	29.5 [19; 34]	−1.65 [−2.33; −0.95]	50	2	32 [30; 34]	−1.5 [−2.05; −0.95]	50
RCFT copy time	8	267.5 [63; 600]	−1.3 [−2.33; −0.95]	25	2	141.5 [128; 155]	−0.95 [−0.95; −0.95]	0
**Visual memory**								
DCS-II LEI	8	26.73 [8.8; 39]	−1.09 [−2.33; 0.03]	37.5	4	28.05 [9.9; 50]	−1.45 [−2.33; −0.05]	50
DCS-II EI	8	36.81 [4.2; 78.2]	−0.64 [−2.33; 1.13]	37.5	4	41.2 [11; 75.5]	−1.5 [−2.33; −0.1]	50
**Executive functions**								
**Verbal fluency**								
Phonemic fluency	6	7.5 [3; 12]	−0.88 [−1.34; 0.67]	0	7	9 [4; 15]	−0.96 [−1.34; 0]	0
**Set-shifting**								
TMT-B	9	113.44 [35; 318]	−0.15 [−1.34; 0.84]	0	7	119 [48; 226]	−0.8 [−1.34; 0.84]	0
**Working memory**								
Digit span backwards	11	5.09 [2; 11]	−0.7 [−2.05; 1.48]	27.3	7	5.57 [3; 8]	−0.46 [−1.75; 1.04]	14.3
**Attention**								
**Processing speed**								
TMT-A	9	34.67 [17; 75]	−0.06 [−1.34; 1.34]	0	7	32.29 [21; 63]	0.18 [−1.34; 1.28]	0
**Attention span**								
Digit span forward	11	6.9 [3; 10]	−0.29 [−2.33; 1.18]	18.2	6	6.67 [6; 8]	−0.48 [−1.13; 0.71]	0

Note: ^**a**^Normative z-scores not available. Percentage of patients with clinical impairment (based on cut-off score z < −1.5). Abbreviations: VLMT = Verbal Learning and Memory Test; ST = short term; LT = long term; DCS-II LEI = Diagnosticum für Cerebralschädigung – II, Learning efficiency index; DCS-II EI = Diagnosticum für Cerebralschädigung – II, Error-Index; RCFT = Rey Complex Figure Test; TMT = Trail Making Test. N/A = Not available.

Neurologically and psychiatrically healthy controls were recruited via several public announcements. Sixteen subjects, matched for age and gender, participated in experiment 1 (six women, mean age ± SD: 51.13 ± 15.48 years, age range: 28–75 years). From this sample, 13 subjects participated in experiment 2 (four women, mean age ± SD: 50.62 ± 15.57 years, age range: 30–75). Nine control subjects were available for FU-measurements of experiment 1; seven of them participated in FU-measurements of experiment 2.

All participants had normal or corrected-to-normal visual acuity and were right-handed (except for one control and three patients). All participants provided written informed consent prior to participation that was in accordance with the Declaration of Helsinki. The study was approved by the ethics committee at the University of Muenster, Germany and conducted accordingly (AZ: 2013-350-f-S and 2013-248-f-S).

### Experimental design

Two established emotion inducing tasks were applied to investigate behavioural and electrodermal emotional responses at BL- and FU-measurements. In experiment 1, participants watched a set of six silent film clips (duration: 40 s) previously described to induce emotional states (i.e. anger, sadness, disgust, fear, and happiness) and a non-emotional neutral state^18^. After each film, participants indicated valence and arousal using nine-point Likert scales, with higher values representing more pleasant or more arousing emotional experiences. A practice clip with neutral content was shown in order to get accustomed to the rating procedure. Stimuli were presented on a 27” computer screen with a resolution of 720 × 576 in a dimly lit room. The inter-stimulus interval was set at 10 s, during which subjects saw a fixation cross, thus allowing electrodermal activity (EDA) to return to baseline^19^.

In experiment 2, stimuli consisted of 30 standardized visual scenes from the IAPS database^20^ that were applied to induce disgust, fear and a non-emotional neutral state. The stimuli have previously been proven useful to induce basic emotional states in a lesion case study by our group^21^. Each of the 30 pictures was presented for 5 s^22^. Presentation was pseudo-randomized, with no repetition of scenes belonging to the same category. Three rating tasks followed each picture. First, subjects were asked to select the emotion that best represented their most prominent emotional sensation to that picture. Percentage match of chosen emotional category with the emotion category intended to be induced (hit rate) served as dependent variable. As in experiment 1, ratings of valence and arousal were recorded. After the ratings, a fixation cross was shown for 10 s before presentation of the next stimulus^19^.

### Skin conductance recording and analysis

EDA was monitored and recorded continuously during both experiments using a BIOPAC MP150 system and the corresponding software AcqKnowledge 4.3. (BIOPAC Systems, Inc.). Presentation software (Neurobehavioral Systems, Inc.) synchronized the physiological monitoring equipment by event markers designating the beginning and ending of each stimulus. Ag/AgCl snap electrodes were used to receive the physiological signal at the thenar and hypothenar eminence of the non-dominant hand, thus allowing for a motor response performed by the dominant hand during the self-report tasks. The room temperature was kept similar for every participant (approx.: 21 °C). Before starting the experimental investigation of emotional processing, a respiration measurement (duration: 60 s) was conducted, during which subjects were asked to remain calm and inhale deeply several times to prove the general ability to develop SCRs. Only after passing this pre-test experimental investigation started.

Processing of electrodermal data was performed with Matlab (MathWorks). A 0.5–2 Hz band-pass filter was applied and data were down-sampled to 250 Hz. For experiment 1, we used the number of extracted galvanic skin responses (nSCR) with a trough-to-peak distance above 0.01 µS^22^ as a measure of EDA for every film clip, in this manner reflecting phasic arousal responses to each kind of emotional stimulation over time^18^. Considering the typical latency of an SCR (1–3 s)^19^, our time window of interest was set to 1–40 s after stimulus onset. For experiment 2, the area under the curve (AUC), a common measure of SCR, was analysed 1–5 s after stimulus onset^19,23^. The first three picture stimuli were excluded to account for habituation. For the remaining nine pictures per category square-root transformation was applied and an average AUC was computed.

### Quantitative high-resolution structural MRI

High-resolution isotropic (1 mm) 3D structural T_1_-weighted imaging data were obtained in all participants using a 3-Tesla system (Magnetom Prisma; Siemens, Erlangen, Germany); in patients, T_2_-weighted and FLAIR sequences were additionally obtained. Volumetric analysis was performed by employing the recon-all script^24,25^ from the FreeSurfer image analysis suite (https://surfer.nmr.mgh.harvard.edu/) on the structural T_1_-weighted images. The recon-all script automatically segments and parcels the whole grey matter and, thus, computes statistical values for each derived region and the intracranial volume. Statistical analyses were based on normalized amygdala volume, i.e. individual amygdala volume divided by individual intracranial volume. MRI data from ten patients and 14 controls were analysed at BL, and MRI data from five patients and nine controls were available at FU.

### Statistical procedures

For each experiment, a mixed factorial ANOVA was performed with the within-subjects factor Emotion (experiment 1: anger, fear, happiness, sadness, disgust, and neutral; experiment 2: fear, disgust, and neutral), and the between-subject factor Group (patients, controls). EDA measures (experiment 1: nSCR; experiment 2: AUC) or rating data (experiment 1: arousal, valence; experiment 2: arousal, valence, hit rate) served as dependent variables. Whenever the assumption of sphericity was violated, the Greenhouse-Geisser correction was applied and $\hat{{\rm{\varepsilon }}}$-values as well as corrected *p*-values are accordingly reported. Significant group effects or interactions were further investigated via planned one-sided two-sample *t*-tests. Relationships between amygdala volume and behavioural resp. autonomic responses were investigated using Pearson’s product-moment correlations. Statistical significance was assumed when *p* < 0.05.

Potential immunotherapeutic effects on emotional processing were investigated for behavioural, EDA and anatomical measures revealing significant group differences in BL-related analyses. First, the difference between FU and BL (difference = value_FU_ – value_BL_) was calculated for behavioural, EDA and anatomic measures; for measures obtained in both experiments (i.e. behavioural and EDA measures) differences were averaged for every subject to form a singular and more reliable index. For patients, these differences were z-transformed (*z*_patient_ = (difference_patient_ – mean difference_controls_)/SD_controls_), thus establishing relations to the distribution of the control group. For the EDA measurements, z-standardization was performed in advance to the averaging in order to account for the different measurement units (i.e. nSCR, AUC). *Z*-values beyond the 95% confidence interval of the control group (i.e. |*z*_patient_| ≥ 1.96) were considered significantly abnormal^18^. In order to investigate changes in neuropsychological data from BL to FU, a Χ²-test was administered.

### Data availability

The datasets generated during and/or analysed during the current study are partially included in this article (Supplementary Material) or are available from the corresponding author on request.

## Results

### Behavioural results at baseline

#### Neuropsychological profile of the patient sample

Most prominent impairments were observed for the verbal and visual memory domain, including reductions of verbal span, verbal learning abilities, verbal short-term and long-term retrieval, as well as visuospatial performance (Table 2).

#### Reports of arousal, valence, and emotion category

Due to technical failure in two patients, behavioural results are premised on data from *n* = 11 (experiment 1) resp. *n* = 10 (experiment 2) patients.

**Figure 2:**
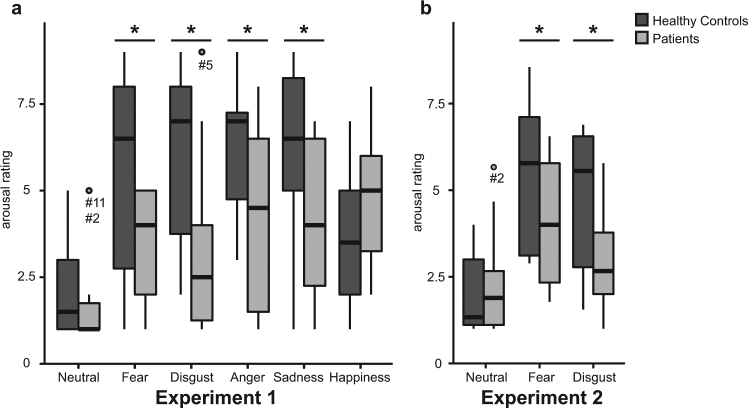
Results for group comparisons of arousal ratings in experiment 1 (**a**) and experiment 2 (**b**). Boxplot diagrams show first and third quartile (bottom and top of the box), the median (second quartile indicated by the band inside the box), and the 1.5 interquartile range (IQR) between the first and third quartile (whiskers). Data points outside of the 1.5 IQR are represented by dots. Statistical analyses revealed significantly diminished arousal ratings in patients as compared to controls for videos (experiment 1) and pictures (experiment 2) aiming to induce negative emotional states.

In experiment 1, ANOVA with arousal ratings as dependent variable yielded a significant main effect of Emotion (*F*(5,120) = 4.81, *p* = 0.002, $\hat{{\rm{\varepsilon }}}$ = 0.78) and a significant Emotion x Group interaction (*F*(5,120) = 13.99, *p* < 0.001, $\hat{{\rm{\varepsilon }}}$ = 0.78). *Post hoc* analyses for the latter effect indicated significantly diminished arousal ratings (all *p < *0.05) in patients as compared to controls for all film clips aiming to induce negative emotional states (i.e. fear, anger, disgust, and sadness) (Fig. 2a). In experiment 2, ANOVA with arousal ratings as dependent variable yielded similar results with a significant main effect of Emotion (*F*(2,40) = 31.64, *p* < 0.001, $\hat{{\rm{\varepsilon }}}$ = 0.74) and a significant Group x Emotion interaction (*F*(2,40) = 4.97, *p* = 0.02, $\hat{{\rm{\varepsilon }}}$ = 0.74). *Post hoc* analyses for the latter effect revealed significantly attenuated arousal ratings for pictures eliciting negative emotional states (i.e. fear and disgust) in patients as related to controls (all *p* < 0.05) (Fig. 2b).

In experiment 1, ANOVA with valence ratings as dependent variable yielded a significant main effect of Emotion (*F*(5,120) = 13.91, *p* < 0.001, $\hat{{\rm{\varepsilon }}}$ = 0.75), while neither the main effect of Group nor the Emotion x Group interaction reached statistical significance (all *p* > 0.50) (Supplementary Fig. 1a). In experiment 2, ANOVA with valence ratings as dependent variable revealed similar results with a significant main effect of Emotion (*F*(2,40) = 53.41, *p < *0.001), but no statistical significance was found for the main effect of Group or the Group x Emotion interaction (all *p* > 0.06) (Supplementary Fig. 1b).

ANOVA with hit rate as dependent variable, an additional dependent measure in experiment 2, indicated a significant main effect of Emotion (*F*(2,40) = 4.17, *p* = 0.03, $\hat{{\rm{\varepsilon }}}$ = 0.76), but neither a significant main effect of Group nor a significant Emotion x Group interaction (all *p* > 0.47) (Supplementary Fig. 1c).

### Autonomic arousal at baseline

**Figure 3:**
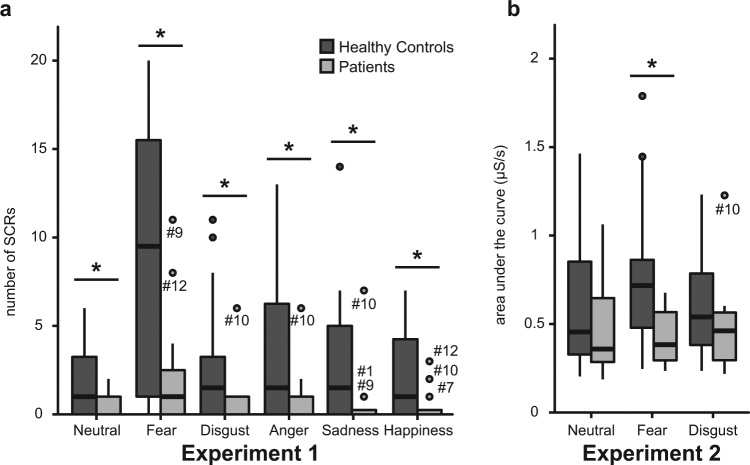
Results for group comparisons of skin conductance measures in experiment 1 (**a**) and experiment 2 (**b**). Boxplot diagrams show first and third quartile (bottom and top of the box), the median (second quartile indicated by the band inside the box), and the 1.5 interquartile range (IQR) between the first and third quartile (whiskers). Data points outside of the 1.5 IQR are represented by dots. Statistical analyses showed significant reductions of autonomic arousal in patients as compared to controls for all emotional conditions of experiment 1, and for the fear-inducing condition of experiment 2.

In experiment 1, ANOVA with nSCR as dependent measure revealed a significant main effect of Emotion (*F*(5,130) = 13.58, *p* < 0.001, $\hat{{\rm{\varepsilon }}}$ = 0.39), a significant main effect of Group (*F*(1,26) = 8.12, *p* = 0.008), and a significant Emotion x Group interaction (*F*(5,130) = 3.67, *p* = 0.03, $\hat{{\rm{\varepsilon }}}$ = 0.39). *Post hoc* analyses indicated significant reductions of autonomic arousal in patients as compared to controls for all emotional conditions (all *p* < 0.05) (Fig. 3a).

In experiment 2, ANOVA with AUC as dependent variable yielded a significant Emotion x Group interaction (*F*(2,44) = 5.63, *p* = 0.01, $\hat{{\rm{\varepsilon }}}$ = 0.78), but neither a significant main effect of Emotion nor a significant main effect of Group (all *p* > 0.09). *Post hoc* analyses indicated significantly attenuated autonomic arousal in patients as compared to controls for the fearful (*p* = 0.01), but not for the neutral or the disgust condition (all *p* > 0.17) (Fig. 3b).

### Correlational results for amygdala volume, autonomic arousal and report of arousal at baseline

**Figure 4:**
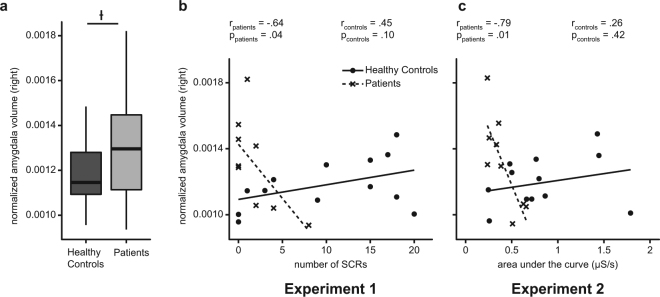
Results from group comparison of normalized right amygdala volume as visualized by means of boxplots (**a**) and correlation analyses, showing significant negative associations between measures of skin conductance from experiment 1 (**b**) resp. experiment 2 (**c**) and normalized right amygdala volume in patients, but not in controls. Boxplot diagrams show first and third quartile (bottom and top of the box), the median (second quartile indicated by the band inside the box), and the 1.5 interquartile range (IQR) between the first and third quartile (whiskers).

When compared to controls, patients showed a marginal significant increase of normalized right (*t*(22) = −1.50, *p* = 0.078), but not left (*t*(22) = −1.24, *p* = 0.120) amygdala volume. As can be seen in Fig. 4a, right amygdala enlargement varied greatly among the patients (from 0.0009 to 0.0018; for details see Supplementary Table 1), which allowed us to examine whether normalized right amygdala volume predicted behavioural and autonomic responses in patients. Since fear-inducing conditions consistently revealed significant differences between patients and controls regarding subjective report and autonomic response in both experiments, associations between normalized right amygdala volume and measures of skin conductance resp. arousal ratings were analysed for fear-inducing stimuli only.

In patients, a significant negative correlation between right amygdala volume and the nSCR (*r* = −0.64, *p* = 0.04, Fig. 4b) resp. AUC (*r* = −0.79, *p* = 0.01, Fig. 4c) was observed. Analogous computations for healthy controls did not reach significance (all *p* > 0.10) and showed, if at all, rather a trend of positive correlations (Fig. 4b,c). The unique association pattern observed in patients suggests that the extent of right amygdala enlargement predicts the deficits revealed by measures of autonomic response to emotional stimulation.

Negative correlations between arousal ratings and normalized right amygdala volume in patients did not reach statistical significance (all *p* > 0.18, not shown), suggesting that autonomic measures are a better indicator of functional disturbance of the amygdala in TLE-AE. Controls showed neither a trend of negative correlations nor statistically significant correlations between arousal ratings and right amygdala volume at all (all *p* > 0.30, not shown). Furthermore, there were no significant correlations for the left amygdala volume with measures of autonomic arousal or arousal ratings in patients or controls (all *p* > 0.05, not shown).

#### Comparative results of patients with confirmed and suspected TLE-AE

In order to address the different serostatus of our patient group, we compared patients with known autoantibodies (seropositive; *n* = 7) and patients without known autoantibodies (seronegative; *n* = 5) on measures that had been found to discriminate between TLE-AE patients and controls. The patient groups were not different on arousal ratings of fearful stimuli (experiment 1: *t*(8) = −1.36, *p* = 0.22, experiment 2: *t*(7) = −1.99, *p* = 0.09). Moreover, the patients did not differ on the autonomic processing of fearful stimuli (experiment 1: *t*(10) = −0.01, *p* = 0.99; experiment 2: *t*(9) = 0.25, *p* = 0.81). In addition, excluding patients without known autoantibodies from correlational analyses did not change the effects reported above (experiment 1: *r* = −0.69, *p* = 0.045, one-sided; experiment 2: *r* = −0.79, *p* = 0.015, one-sided).

### Treatment effects

#### Neuropsychological profile

No significant differences were found between BL and FU neuropsychological profiles (all *p* > 0.433) (Table 2).

#### Emotional processing and structural changes at follow-up

**Figure 5:**
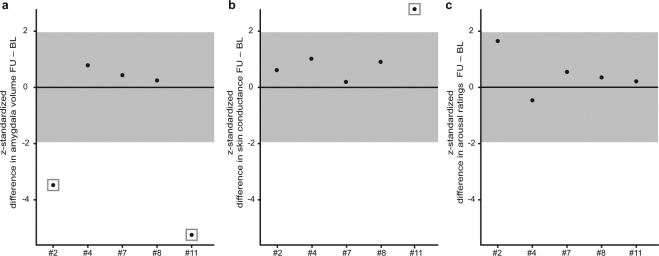
Z-transformed structural (**a**), behavioural (**b**) and autonomic (**c**) changes from baseline (BL) to follow-up (FU) for five TLE-AE patients that were followed during the course of immunotherapy. Zero line represents the control group’s mean, grey zone denotes the 95% confidence interval [−1.96, 1.96]. Significant differences (i.e. |z| ≥ 1.96) are marked by grey squares. Significant reductions of normalized right amygdala volume at FU were registered for patient #2 and patient #11. Patient #11 who suffered from confirmed autoimmune TLE-AE with anti-LGI1 autoantibodies with a very recent clinical onset, thus receiving prompt aggressive immunotherapy, showed a significant increase of autonomic arousal. No behavioural changes were registered.

Analyses of potential immunotherapeutic effects on emotional processing were restricted to fear-inducing conditions, since these revealed the most prominent deficits of autonomic arousal and subjective arousal report in patients. Out of five patients completing experimental procedures at the FU, only patient #11 who suffered from confirmed autoimmune TLE-AE with anti-LGI1 antibodies with a very recent clinical onset (disease duration of 1 month) and, thus, received prompt aggressive immunotherapy consisting of IA, MPPT, and RTX, showed a significant increase of autonomic arousal (but no significantly altered arousal ratings) (*z*_11_ = 2.79, *p* = 0.005) (Fig. 5b,c). Concerning anatomical changes, patient #11 also showed the highest decrease in right amygdala volume after immunotherapy as compared to controls (*z*_11_ = −5.25, *p* < 0.001) (Fig. 5a). Only one further patient (patient #2) showed a significant decrease in right amygdala volume after immunotherapy (*z*_2_ = −2.79, *p* = 0.005).

## Discussion

In the present study, we examined behavioural and autonomic responses to emotional stimulation in a sample of treatment-naïve patients suffering from suspected or confirmed autoimmune TLE-AE in relation to matched healthy controls. We found markedly reduced arousal ratings and autonomic responsivity to emotionally arousing stimulation in TLE-AE, being mostly pronounced for anxiety-inducing stimulation. The right amygdala volume was significantly predictive of EDA attenuation in patients. A decline of amygdala swelling after immunotherapy was accompanied by restored autonomic responses in one patient who suffered from severe confirmed autoimmune TLE-AE with a very recent clinical onset and, thus, was treated with prompt aggressive immunotherapy.

The pattern of attenuated autonomic responses to emotional stimulation observed in the current sample of patients corresponds with the almost absent emotional modulation of autonomic responses previously described in an immunotherapy-naïve patient suffering from confirmed autoimmune TLE-AE with anti-CASPR2 aabs^18^. Our group study shows that these deficits seem to be related to the prominent amygdala involvement in TLE-AE. This conclusion is substantiated by our correlation findings, showing that the degree of right amygdala enlargement predicted SCR reductions in fear-inducing conditions. The amygdala is involved in stimulus relevance appraisal and induction of appropriate autonomic responses via reciprocal connections of centromedial amygdaloid nuclei to the autonomic centers of the brain stem and hypothalamus^26^. Being one of the key regions for generating and modulating autonomic tone^27^, the amygdala is implied to exert direct and excitatory influence on SCR generation and amplitude^28^. Interestingly, left and right amygdalae are suggested to subserve different, but complementary affective information-processing functions. While the right amygdala is emphasized in the rapid and automatic detection of any arousing stimulus (e.g. a signal of potential threat) and the subsequent induction of a general level of physiological arousal contributing to dynamic emotional stimulus detection, the left amygdala is regarded to mediate the detailed and sustained stimulus evaluation by discerning specific differences in arousal magnitude, thus contributing to more cognitive perceptual emotional information processing^23,29^. The significant correlation between the degree of right, but not left, amygdala enlargement and attenuated EDA in our study might be explained by the delineated functional lateralization, emphasizing the right amygdala as the primary basis of emotion-related physiological responses.

Analyses of behavioural emotional responses revealed deficits of arousal, but not of valence or emotion ratings in TLE-AE patients. This finding corresponds with other studies showing impaired cognitive and autonomic indices of arousal processing but intact recognition of emotional valence after various amygdala pathologies^23,30^. Interestingly, our study shows that while amygdala volume was significantly associated with EDA attenuation in TLE-AE, there was no such relationship for deficits in arousal ratings. This suggests that autonomic changes are a better predictor for amygdala alterations in TLE-AE. However, since the correlations between amygdala volume and arousal ratings showed a negative trend across both designs, future studies with increased sample size might further investigate the strength of the relation between structural changes of the amygdala and cognitive arousal measures.

We show at least tentative evidence that amygdala enlargement and impairments of emotional physiological responses might be reduced after immunotherapy. One patient suffering from TLE-AE with anti-LGI1 antibodies (known to at least partially recover after immunotherapy^31,32^) with the shortest disease duration (1 month) promptly received the most aggressive immunotherapy among all patients consisting of IA, MPPT, and RTX (leading to immediate B cell depletion) at BL. At FU, this patient showed the most pronounced reduction of amygdala volume and also improved emotional physiological responses. This finding is in very good agreement with recent data showing improved cognitive outcomes with early effective immunotherapy in anti-LGI1 encephalitis^33^. Effects of early and rapidly effective immunotherapies should be considered in future research with samples of recent-onset aab-confirmed autoimmune TLE-AE patients.

Some limitations should be mentioned. Although examining a high number of patients (given the clinical rareness of TLE-AE), the underlying autoimmune aetiology remained suspected in five patients. At this point we could not formally exclude focal cortical dysplasia or tumors confined to the enlarged amygdalae as the underlying aetiology especially in two aab-negative patients with unilateral AE (patients #1 and #4) despite inflammatory CSF changes seen in all patients. However, complementary analysis revealed no behavioural and autonomic differences between patients with confirmed and suspected TLE-AE when processing fearful stimuli. Moreover, the negative correlation between right amygdala volume and SC measures did not change when excluding patients with suspected TLE-AE. Notwithstanding these results, future studies should include larger cohorts of recent-onset treatment-naive aab-confirmed autoimmune TLE-AE patients of all subtypes to confirm the independence of reduced behavioural and autonomic emotional responses and their relation to amygdala enlargement from distinct aetiologies.

Concluding, our study shows that patients with TLE-AE develop - besides known impairments in declarative memory functions - pronounced emotional disturbances as reflected in reduced autonomic responsivity and attenuated subjective report of arousal to emotional stimuli. These prominent alterations might be attributed to the extent of amygdala involvement in TLE-AE. Moreover, preliminary evidence suggests that successful immunotherapy in autoimmune TLE-AE might not only be accompanied by reduced amygdala enlargement but also by recovery of impaired autonomic emotional responses.

## Electronic supplementary material

Supplementary Material
