# Supplementary material for: Amygdala enlargement and emotional responses in (autoimmune) temporal lobe epilepsy
Source: Sci Rep. 2018 Jun 22;8:9561. doi: 10.1038/s41598-018-27914-z (PMC6015084; doi:10.1038/s41598-018-27914-z)
Supplement: Supplementary file 1 — Supplementary Material [file 41598_2018_27914_MOESM1_ESM.docx]

**Amygdala enlargement and emotional responses in (autoimmune) temporal lobe epilepsy**

**Running head:** Emotion processing in TLE-AE

**Authors:** Olga Holtmann^1,2§*,^, Insa Schlossmacher^1,2§^, Constanze Moenig^3§^, Andreas Johnen^3^, Lisa-Marie Rutter^3^, Jan-Gerd Tenberge^3^, Patrick Schiffler^3^, Judith Everding^1^, Kristin S. Golombeck^3^, Christine Strippel^3^, Andre Dik^3^, Wolfram Schwindt^4^, Heinz Wiendl^3^, Sven G. Meuth^3^, Maximilian Bruchmann^1,2^, Nico Melzer^3§^, Thomas Straube^1,2§^

^1^ Institute of Medical Psychology and Systems Neuroscience, University of Muenster,

Muenster, Germany

^2^ Otto Creutzfeldt Center for Cognitive and Behavioral Neuroscience, University of Muenster, Muenster, Germany

^3^ Department of Neurology, University of Muenster, Muenster, Germany

^4^ Department of Clinical Radiology, University of Muenster, Muenster, Germany

^§^contributing equally

**Supplementary Figures**

**
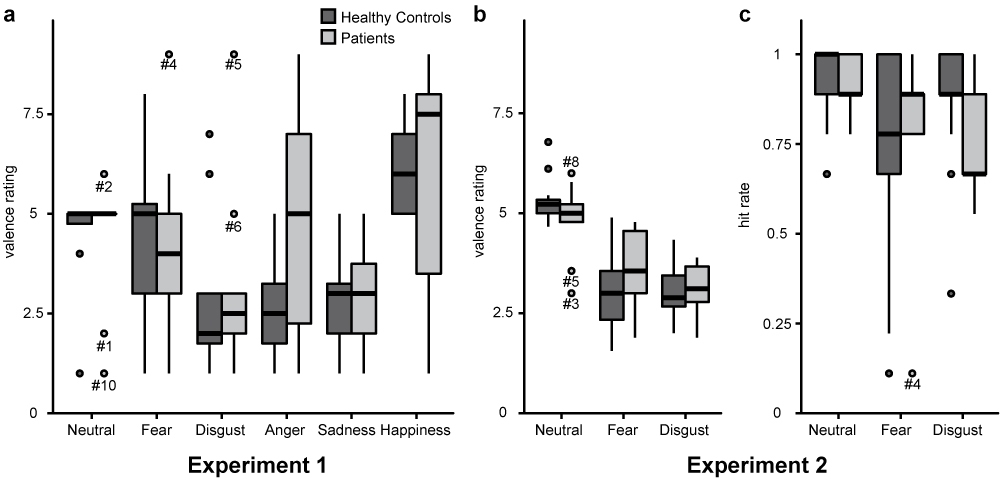
**

**Supplementary Figure 1.** Group comparisons of valence ratings in experiment 1 (a) and experiment 2 (b), as well as hit rate in experiment 2 (c) did not reveal any significant differences between patients and controls. Boxplot diagrams show first and third quartile (bottom and top of the box), the median (second quartile indicated by the band inside the box), and the 1.5 interquartile range (IQR) between the first and third quartile (whiskers).

**Supplementary Tables**

**Supplementary Table 1. Structural volumes at baseline and follow-up**

|  | **Baseline** | | | | |  | **Follow-up** | | | | | |
| --- | --- | --- | --- | --- | --- | --- | --- | --- | --- | --- | --- | --- |
| **Patient** | **total intra-cranial volume** | **right amygdala** | **left amygdala** | **right hippocampus** | **left hippocampus** |  | **month after baseline** | **total intra-cranial volume** | **right amygdala** | **left  amygdala** | **right hippocampus** | **left hippocampus** |
| #1 | 1397730 | 1811.8 | 2100.6 | 4496.8 | 4275.8 |  | - | - | - | - | - | - |
| #2 | 1435289 | 2219.8 | 1787.2 | 4553.3 | 4078.2 |  | 3 | 1440656 | 1691.0 | 1587.9 | 4032.6 | 4239.1 |
| #3 | 1535301 | 2237.1 | 2241.7 | 5463.5 | 5312.3 |  | - | - | - | - | - | - |
| #4 | 1442253 | 1855.2 | 2041.8 | 5005.1 | 5044.2 |  | 6 | 1428997 | 1943.9 | 1903.2 | 5055.5 | 4836.8 |
| #5 | - | - | - | - | - |  | - | - | - | - | - | - |
| #6 | 1453138 | 2058.5 | 1537.5 | 4265.8 | 4544.4 |  | - | - | - | - | - | - |
| #7 | 1706288 | 1802.1 | 2249.6 | 4333.5 | 5130.6 |  | 6 | 1699452 | 1858.3 | 2392.9 | 4188.9 | 4892.4 |
| #8 | 1580898 | 2047.7 | 1446.3 | 4525.2 | 4871.2 |  | 3 | 1578723 | 2072.9 | 1555.3 | 4678.3 | 4570.0 |
| #9 | - | - | - | - | - |  | - | - | - | - | - | - |
| #10 | 1657332 | 1723.7 | 1452.7 | 4478.3 | 4088.8 |  | - | - | - | - | - | - |
| #11 | 1236514 | 2251.4 | 2423.5 | 5366.2 | 6181.2 |  | 3 | 1270118 | 1603.0 | 2169.6 | 4960.2 | 4677.5 |
| #12 | 1556087 | 1456.7 | 2065.7 | 4012.5 | 4613.4 |  | - | - | - | - | - | - |
| Patients, mean (SD) | 1500083  (136563.8) | 1946.4 (260.7) | 1934.7 (355.7) | 4650.0  (475.3) | 4814.0 (645.0) |  |  | 1483589 (162819.5) | 1833.8 (189.4) | 1921.8 (363.8) | 4583.1 (456.3) | 4643.2 (259.5) |
| Controls, mean (SD) | 1462967 (118781.4) | 1718.0 (251.8) | 1723.8 (270.4) | 4491.8 (481.5) | 4347.1 (574.3) |  |  | 1500386 (93619.9) | 1734.7 (272.9) | 1790.6 (321.0) | 4601.4 (521.8) | 4469.9 (559.9) |

*Note.* All structural data are measured in mm^3^.

**Supplementary Table 2. Arousal ratings at baseline and follow-up**

|  | **Baseline** | | | | | | | | | |  | **Follow-up** | | | | | | | | | |
| --- | --- | --- | --- | --- | --- | --- | --- | --- | --- | --- | --- | --- | --- | --- | --- | --- | --- | --- | --- | --- | --- |
|  | **Experiment 1** | | | | | |  | **Experiment 2** | | |  | **Experiment 1** | | | | | |  | **Experiment 2** | | |
| **Patient** | **Neutral** | **Fear** | **Disgust** | **Anger** | **Sadness** | **Happiness** |  | **Neutral** | **Fear** | **Disgust** |  | **Neutral** | **Fear** | **Disgust** | **Anger** | **Sadness** | **Happiness** |  | **Neutral** | **Fear** | **Disgust** |
| #1 | 2 | 2 | 4 | 8 | 7 | 7 |  | - | - | - |  | - | - | - | - | - | - |  | - | - | - |
| #2 | 5 | 1 | 3 | 5 | 1 | 3 |  | 5.67 | 4.67 | 2.33 |  | 5 | 5 | 7 | 5 | 9 | 6 |  | 4 | 6.67 | 6.89 |
| #3 | 1 | 1 | 1 | 1 | 2 | 3 |  | 1.67 | 1.78 | 2 |  | - | - | - | - | - | - |  | - | - | - |
| #4 | 1 | 2 | 1 | 1 | 1 | 2 |  | 1.11 | 2.33 | 1.56 |  | 1 | 1 | 7 | 1 | 2 | 5 |  | 1 | 1.89 | 1 |
| #5 | 1 | 5 | 9 | 7 | 5 | 6 |  | 2 | 4 | 2.67 |  | - | - | - | - | - | - |  | - | - | - |
| #6 | 1 | 5 | 2 | 5 | 4 | 5 |  | 1.89 | 6.56 | 5.78 |  | - | - | - | - | - | - |  | - | - | - |
| #7 | 1 | 5 | 1 | 1 | 7 | 4 |  | 1 | 5.78 | 3.67 |  | 1 | 7 | 1 | 2 | 9 | 2 |  | 2.44 | 5.44 | 2.67 |
| #8 | 1 | 5 | 2 | 3 | 3 | 6 |  | 1 | 2.22 | 1 |  | 2 | 6 | 4 | 5 | 5 | 5 |  | 1 | 2.11 | 1.33 |
| #9 | - | - | - | - | - | - |  | - | - | - |  | - | - | - | - | - | - |  | - | - | - |
| #10 | 1 | 5 | 7 | 7 | 7 | 8 |  | 2.67 | 5.78 | 5.33 |  | - | - | - | - | - | - |  | - | - | - |
| #11 | 5 | 3 | 4 | 4 | 4 | 5 |  | 4.67 | 2.89 | 3.78 |  | 3 | 4 | 2 | 3 | 3 | 4 |  | 1.44 | 2.22 | 2.44 |
| #12 | - | - | - | - | - | - |  | - | - | - |  | - | - | - | - | - | - |  | - | - | - |
| Patients, mean (SD) | 1.9 (1.66) | 3.4 (1.78) | 3.4 (2.71) | 4.2 (2.66) | 4.1 (2.38) | 4.9 (1.91) |  | 2.41 (1.67) | 4.00 (1.78) | 3.12 (1.65) |  | 2.4 (1.67) | 4.6 (2.30) | 4.2 (2.77) | 3.2 (1.79) | 5.6 (3.29) | 4.4 (1.52) |  | 1.98 (1.28) | 3.49 (2.43) | 3.04 (2.21) |
| Controls, mean (SD) | 2.13 (1.45) | 5.56 (2.83) | 6.13 (2.47) | 6.19 (2.14) | 6.13 (2.45) | 3.56 (1.86) |  | 2.09 (1.15) | 5.46 (2.17) | 4.82 (1.97) |  | 1.89 (1.69) | 5.56 (3.17) | 6.67 (2.60) | 5.56 (3.24) | 5.89 (2.71) | 2.56  (1.88) |  | 1.82 (0.88) | 5.03 (2.42) | 5.10 (2.25) |

*Note.* All rating data were measured on a 9-point Likert-scale with higher ratings representing higher arousal.

**Supplementary Table 3. Autonomic measures at baseline and follow-up**

|  | **Baseline** | | | | | | | | | |  | **Follow-up** | | | | | | | | | |
| --- | --- | --- | --- | --- | --- | --- | --- | --- | --- | --- | --- | --- | --- | --- | --- | --- | --- | --- | --- | --- | --- |
|  | **Experiment 1** | | | | | |  | **Experiment 2** | | |  | **Experiment 1** | | | | | |  | **Experiment 2** | | |
| **Patient** | **Neutral** | **Fear** | **Disgust** | **Anger** | **Sadness** | **Happiness** |  | **Neutral** | **Fear** | **Disgust** |  | **Neutral** | **Fear** | **Disgust** | **Anger** | **Sadness** | **Happiness** |  | **Neutral** | **Fear** | **Disgust** |
| #1 | 0 | 0 | 0 | 0 | 1 | 0 |  | - | - | - |  | - | - | - | - | - | - |  | - | - | - |
| #2 | 0 | 0 | 0 | 0 | 0 | 0 |  | 0.34 | 0.36 | 0.35 |  | 0 | 0 | 0 | 0 | 0 | 0 |  | 0.26 | 0.27 | 0.28 |
| #3 | 0 | 0 | 0 | 0 | 0 | 0 |  | 0.25 | 0.26 | 0.23 |  | - | - | - | - | - | - |  | - | - | - |
| #4 | 0 | 0 | 0 | 0 | 0 | 0 |  | 0.36 | 0.38 | 0.22 |  | 1 | 3 | 1 | 0 | 0 | 1 |  | 0.41 | 0.44 | 0.44 |
| #5 | 1 | 1 | 0 | 0 | 0 | 0 |  | 0.75 | 0.49 | 0.60 |  | - | - | - | - | - | - |  | - | - | - |
| #6 | 0 | 2 | 0 | 1 | 0 | 0 |  | 0.24 | 0.33 | 0.33 |  | - | - | - | - | - | - |  | - | - | - |
| #7 | 1 | 2 | 1 | 0 | 0 | 1 |  | 0.43 | 0.63 | 0.54 |  | 2 | 0 | 0 | 0 | 0 | 1 |  | 0.28 | 0.35 | 0.31 |
| #8 | 0 | 0 | 0 | 0 | 0 | 0 |  | 0.19 | 0.24 | 0.26 |  | 0 | 3 | 0 | 2 | 0 | 1 |  | 0.27 | 0.22 | 0.27 |
| #9 | 0 | 11 | 1 | 2 | 1 | 0 |  | 0.72 | 0.68 | 0.59 |  | - | - | - | - | - | - |  | - | - | - |
| #10 | 1 | 4 | 6 | 6 | 7 | 2 |  | 1.06 | 0.66 | 1.23 |  | - | - | - | - | - | - |  | - | - | - |
| #11 | 0 | 1 | 1 | 0 | 0 | 0 |  | 0.32 | 0.24 | 0.46 |  | 6 | 6 | 10 | 1 | 5 | 8 |  | 1.43 | 1.30 | 1.20 |
| #12 | 2 | 8 | 0 | 1 | 0 | 3 |  | 0.58 | 0.51 | 0.53 |  | - | - | - | - | - | - |  | - | - | - |
| Patients, mean (SD) | 0.42 (0.67) | 2.42 (3.58) | 0.75 (1.71) | 0.83 (1.75) | 0.75 (2.01) | 0.5 (1) |  | 0.48 (0.27) | 0.43  (0.17) | 0.49 (0.28) |  | 1.80 (2.49) | 2.40 (2.51) | 2.20 (4.38) | 0.60 (0.89) | 1.00 (2.24) | 2.20  (3.27) |  | 0.53 (0.51) | 0.52 (0.45) | 0.50 (0.40) |
| Controls, mean (SD) | 1.88 (1.96) | 9.13 (7.69) | 2.94 (3.62) | 3.56 (4.13) | 3.13 (3.79) | 2.19 (2.71) |  | 0.61 (0.37) | 0.80 (0.48) | 0.60 (0.31) |  | 1.56 (2.65) | 7.44 (7.80) | 2.44 (2.46) | 2.73 (3.64) | 2.44 (3.13) | 1.78 (2.64) |  | 0.65 (0.46) | 0.60 (0.38) | 0.65 (0.40) |

*Note.* Autonomic measures were collected as nSCR for experiment 1 and AUC for experiment 2
